# Two Coregulated Efflux Transporters Modulate Intracellular Heme and Protoporphyrin IX Availability in *Streptococcus agalactiae*

**DOI:** 10.1371/journal.ppat.1000860

**Published:** 2010-04-22

**Authors:** Annabelle Fernandez, Delphine Lechardeur, Aurélie Derré-Bobillot, Elisabeth Couvé, Philippe Gaudu, Alexandra Gruss

**Affiliations:** 1 Institut National de la Recherche Agronomique, UMR1319 Micalis, Bâtiment 222, Domaine de Vilvert, Jouy-en-Josas, France; 2 Institut Pasteur, Laboratoire Evolution et Génomique Bactérienne, CNRS URA 2171, Paris, France; Children's Hospital Boston, United States of America

## Abstract

*Streptococcus agalactiae* is a major neonatal pathogen whose infectious route involves septicemia. This pathogen does not synthesize heme, but scavenges it from blood to activate a respiration metabolism, which increases bacterial cell density and is required for full virulence. Factors that regulate heme pools in *S. agalactiae* are unknown. Here we report that one main strategy of heme and protoporphyrin IX (PPIX) homeostasis in *S. agalactiae* is based on a regulated system of efflux using two newly characterized operons, *gbs1753 gbs1752* (called *pefA pefB*), and *gbs1402 gbs1401 gbs1400* (called *pefR pefC pefD*), where *pef* stands for ‘porphyrin-regulated efflux’. *In vitro* and *in vivo* data show that PefR, a MarR-superfamily protein, is a repressor of both operons. Heme or PPIX both alleviate PefR-mediated repression. We show that bacteria inactivated for both Pef efflux systems display accrued sensitivity to these porphyrins, and give evidence that they accumulate intracellularly. The Δ*pefR* mutant, in which both *pef* operons are up-regulated, is defective for heme-dependent respiration, and attenuated for virulence. We conclude that this new efflux regulon controls intracellular heme and PPIX availability in *S. agalactiae*, and is needed for its capacity to undergo respiration metabolism, and to infect the host.

## Author Summary

The infectious route of numerous bacterial pathogens includes septicemia, where bacteria are exposed to heme-rich blood. Heme (iron protoporphyrin IX) is generally considered a bacterial iron source. However, while some pathogens do not biosynthesize heme, they use environmental heme to activate key functions. For example, incorporation of heme by the major neonatal pathogen *Streptococcus agalactiae* activates a latent respiration chain. Respiration metabolism stimulates *S. agalactiae* growth and survival in blood, and is needed for virulence. While the importance of heme in *S. agalactiae* behavior is documented, how it manages its intracellular heme pools remains unknown. We discovered a novel regulon, called Pef for “porphyrin-regulated efflux”, that modulates *S. agalactiae* intracellular availability of heme and protoporphyrin IX. A single transcriptional regulator, PefR, represses two distinct efflux transport operons. Regulator-mediated repression is alleviated by heme or protoporphyrin IX. Importantly, over-expression of Pef efflux transporters led to intracellular heme insufficiency, and consequent respiration and virulence defects. Inversely, when Pef efflux transporters were inactivated, results indicated an increased intracellular accumulation of heme and protoporphyrin IX. These studies point to the important role of regulated efflux transport systems in bacterial pathogens for maintaining intracellular heme at levels sufficient to stimulate growth and promote infection.

## Introduction

Heme (iron protoporphyrin IX) is a redox-active molecule, and a cofactor for numerous cell functions used in oxygen sensing and signal transmission, metabolism, and metal homeostasis [1] [–] [3]. In addition to its varied activities as a cofactor, heme promotes toxic oxygen radical production [4]. The duality between heme as a multifunctional cofactor, and a potentially toxic molecule, suggests the need for strict limitation of its intracellular levels. Metal-free protoporphyrin IX (PPIX) is also found intracellularly, as a heme precursor in bacteria that synthesize heme, and as an intermediate during iron recovery from heme, as shown in *Escherichia coli* [5]. Thus, cells might have to deal with both heme and PPIX intracellular pools.

While most studied organisms synthesize heme to ensure activity of hemoproteins under the appropriate conditions, numerous bacteria lack heme biosynthesis genes, making heme-catalyzed processes fully dependent upon external heme supplies. For example, *Haemophilus influenzae*, *Bacteroides* sp., and several Firmicutes, including *Lactococcus lactis*, *Enterococcus faecalis*, *Streptococcus agalactiae*, and numerous *Lactobacillus* sp., require heme to activate a respiration metabolic pathway [6] [18]. Some of these bacteria (e.g., *H. influenzae*, *E. faecalis*, *Bacteroides* sp., *Lactobacillus brevis* and *Lactobacillus plantarum*) also encode heme-dependent catalases, which rely on exogenous heme for both their stability and activity [19]. Thus, heme supplied by the environment can have a determining effect on the metabolic and enzymatic capacities in organisms lacking the heme biosynthetic pathway.

All bacteria, regardless of their heme biosynthesis capacities, need to manage their intracellular heme pools. Regulation may be exerted at the levels of biosynthesis, uptake, degradation, and possibly efflux, which is in some cases coordinated with the cell iron status [20] [25]. The use of efflux as a means of heme homeostasis remains in question. The sole candidate, HrtAB, a heme-regulated transporter, was first reported as having a role in heme toxicity in *Staphylococcus aureus*; orthologs of this system were also described in *L. lactis* and *Bacillus anthracis* [12], [26] [–] [28]. HrtAB in *S. aureus*, *B. anthracis*, and likely several Gram-positive pathogens responds to an extracytoplasmic heme sensor, HssS (part of HssSR two-component system) to activate expression [27], [28].

*S. agalactiae* is an important human pathogen that does not synthesize its own heme. Nevertheless, infection by this bacterium involves compulsory passage through the bloodstream, causing septicemia and subsequent meningitis [29], [30]. We showed previously that although *S. agalactiae* generally grows by a fermentation metabolism, it can also use heme, present in blood, to activate the terminal cytochrome *bd* quinol oxidase for respiration metabolism. Thus, beyond the potential use of heme uptake to acquire iron, the use of heme to activate the energetically favorable respiration metabolism confers a significant gain in cell density, and is required for full bacterial virulence in a septicemia model [17], [18].

Despite the importance of growth in heme-rich blood as an initial step of *S. agalactiae* infection, the functions involved in regulating intracellular heme levels are unknown. Homologs of HrtAB and HssSR exist in *S. agalactiae*, and might participate in modulating heme toxicity upon sensing of extracellular heme. However, the mechanism by which HrtAB modulates heme toxicity is unknown, and the question remains whether other systems are needed to regulate heme pools. Here we report the existence of a novel regulon comprising two efflux operons and a single repressor that senses intracellular heme and PPIX, and is needed to maintain their homeostasis. This newly described system is shown to impact on *S. agalactiae* respiration capacity and virulence.

## Results

### Identification of *gbs1753 gbs1752* as a putative heme- and PPIX- induced locus in *S. agalactiae*

**Figure 1:**
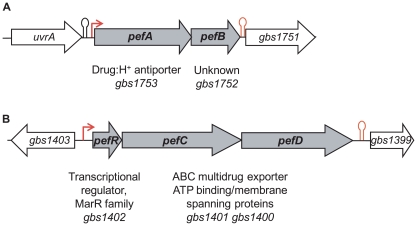
Genetic context of two loci for which expression is heme-induced. **A**, *gbs1753 gbs1752* (*pefA pefB*) and **B**, *gbs1402 gbs1401 gbs1400* (*pefR pefC pefD*) loci. Bent arrows and lollipops indicate the mapped or putative promoters, and rho-independent terminators, respectively.

Transcriptome studies, comparing *S. agalactiae* gene expression under respiration (i.e., aerobic growth in the presence of exogenous heme and menaquinone) *versus* aerobic fermentation (i.e., no addition) conditions, initially revealed that *gbs1753 gbs1752* was 2.4 to 5.1 fold higher under respiration conditions (AD, PG, EC, and P. Glaser [Pasteur Institute]; Table S1). The *gbs1753* ORF encodes an integral membrane protein of the drug:H+ antiporter family, belonging to the major facilitator superfamily (MFS) [31]. The *gbs1752* ORF encodes an unknown protein with 2 transmembrane domains. We chose this locus for study, as it was conserved among Gram-positive bacteria that lack a complete heme biosynthesis pathway, but encode cytochrome *bd* quinol oxidases, indicating a capacity for respiration metabolism (Fig. 1 and Table S2).

**Figure 2:**
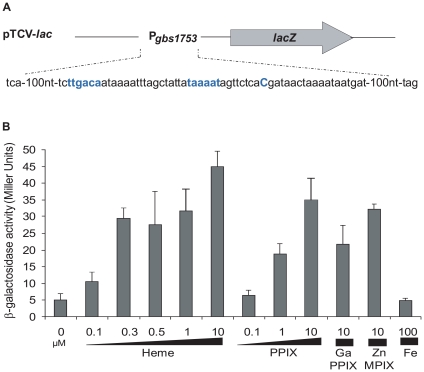
The *pefAB* locus is induced by porphyrin molecules: heme, PPIX, GaPPIX and ZnMPIX. **A**. Schematic map of the transcriptional fusion P*_pefA_-lacZ* (same as P*_gbs1753_-lacZ*). Sequence of the *pefAB* promoter region is displayed. The +1 transcriptional start and −10 and −35 motifs are in bold blue characters. **B**. Expression analysis of P*_pefA_-lacZ* by determination of β-galactosidase activity in early stationary phase. *S. agalactiae* was grown in BHI liquid medium in the presence of the indicated amount of candidate inducers. Strains harboring the pTCV-*lac* vector (promoterless negative control) did not show β-galactosidase activity during growth (0.4±0.1 Miller Units). Measurements were performed at least three times.

The +1 transcriptional start of *gbs1753 gbs1752* was located by 5′-RACE PCR mapping at 134 nucleotides upstream of the *gbs1753* start codon at a cytosine (Fig. 2A). The *gbs1753* promoter region was fused to a *lacZ* reporter, referred to as P*_gbs1753_-lacZ*, and expressed on a low copy number plasmid (Fig. 2A) [32]. In the absence of added heme, P*_gbs1753_-lacZ* displayed basal level expression in static and aerobic growth conditions (possibly reflecting trace amounts of heme in BHI medium). Cultures grown with added heme (from 0.1 µM to 10 µM) displayed up to 9-fold higher P*_gbs1753_-lacZ* expression compared to controls without heme, even in non-respiration-permissive conditions (Fig. 2B), indicating that heme, and not the state of respiration, was the inducing factor for the *gbs1753* promoter. Significant P*_gbs1753_-lacZ* induction was observed at heme concentrations of 0.3 µM and above. Similarly, PPIX, gallium protoporphyrin (GaPPIX), and zinc mesoporphyrin (ZnMPIX) also induced P*_gbs1753_-lacZ*, while free iron had no inducing effect. These initial results indicate that *gbs1753* expression is induced by different porphyrin molecules, regardless of their metal core status.

### Identification of Gbs1402 as a putative regulator of *gbs1753 gbs1752* expression

A random mutagenesis approach was used to identify genetic factors that affect *gbs1753 gbs1752* expression. A transposon-generated mutant library was screened for clones in which expression of the P*_gbs1753_-lacZ* fusion was up-regulated, as detected by deep blue colony color. Several mutations mapped in a single ORF, *gbs1402*. The *gbs1402* gene encodes a multiple antibiotic resistance MarR-like regulator (for review see [33]), and is located just upstream of *gbs1401 gbs1400*, encoding a putative ABC-type multidrug transport complex (Fig. 1B). Sequence analysis predicted that the Gbs1401 and Gbs1400 proteins both contain a transmembrane domain and an ATPase signature. These observations led us to formulate a working hypothesis that both *gbs1753 gbs1752* and *gbs1401 gbs1400* loci are involved in PPIX and metalloporphyrin efflux, and are regulated by a single protein, Gbs1402. The genes were renamed as *pef* genes, for porphyrin-regulated efflux. *gbs1753 gbs1752* are renamed *pefA pefB*; *gbs1401 gbs1400* are renamed *pefC pefD*; and the *gbs1402* gene for the potential regulator is renamed *pefR* (Fig. 1).

### PefR binds specifically to *pefAB* and *pefRCD* promoter regions and represses their transcription

**Figure 3:**
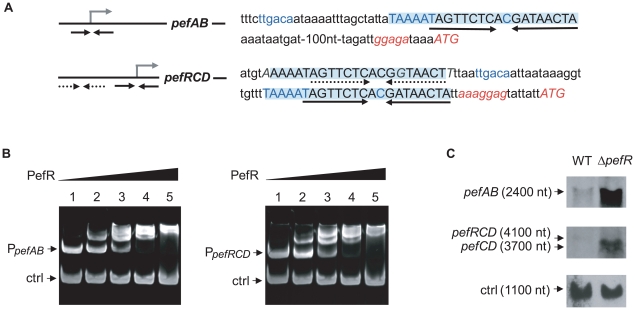
PefR is a repressor of *pefAB* and *pefRCD* loci. **A**. A conserved 23-nucleotide motif is present once upstream of *pefAB*, and twice upstream of *pefRCD* and is highlighted. The IR present in each of the motifs is marked by arrows. The −10 and −35 motifs, and the mRNA *pefAB* and putative *pefRCD* start sites are in blue. Start codons and RBS sequence are in italics. One motif upstream of *pefRCD* (dotted arrows) differs from the other motifs by 3 nucleotides, which are in gray italics. **B**. PefR binds the *pefAB* and *pefRCD* promoter regions in gel shift assays. Two pmoles of *pefAB* or *pefRCD* promoter fragment were incubated in the presence of 0, 4, 17, 34, 63 pmoles of PefR (lanes 1 to 5) and 4.6 pmoles control fragment, corresponding to an 116-bp fragment of the *pefA* gene. **C**. Northern blot analyses of *pefAB* and *pefRCD* mRNA in the WT and *pefR* strains, using locus-specific probes (see Materials and Methods). As probe efficiencies and times of exposure differ for each target RNA, differences between *pefAB* and *pefRCD* levels are not comparable. *ldhL* mRNA (‘ctrl’) was used to control for RNA quantity.

Proteins of the MarR family characteristically bind to DNA inverted repeat motifs (IR) [33]. Sequence analysis revealed the presence of a near-perfect 18-nucleotide IR within a 23 bp consensus, (5′-*T*AAAATAGTTCTCACG*A*TAACT*A*-3′) present once upstream of *pefA pefB*, and twice (one identical sequence, and one inexact copy with 3 nucleotide substitutions in italics) upstream of *pefR pefC pefD* (Fig. 3A). The IR is not present elsewhere on the *S. agalactiae* genome. This 23-nucleotide sequence contains the −10 region (TAAAAT) of a putative promoter and constituted a candidate target site for PefR binding to *pefAB* and *pefRCD* promoter regions. Mobility shift assays were performed using a purified PefR His-fusion protein, in combination with DNA fragments comprising the IRs of *pefAB* or *pefRCD* promoter regions, plus a fragment without an IR as negative internal control (Fig. 3B). PefR caused a mobility shift of both the *pefAB* and *pefRCD* fragments in a protein concentration-dependent manner. No shift was observed with the control DNA fragment.

To determine whether PefR impacts on *pefA pefB* and *pefR pefC pefD* expression *in vivo*, Northern blot experiments were performed on the WT strain and an in-frame Δ*pefR* mutant, using *pefAB* and *pefC* as probes (Fig. 3C). The deduced transcript sizes were compatible with organization of the *pefA* and *pefB* genes as one operon, and of *pefR*, *pefC*, and *pefD* genes as another. Compared to the WT strain, expression of both *pefAB* and *pefRCD* operons was strongly increased in the Δ*pefR* mutant. We also evaluated expression of the P*_pefA_-lacZ* transcriptional fusion in the WT and Δ*pefR* mutant strains. β-galactosidase expression in the Δ*pefR* background was 12 times higher than in the WT strain (Miller Units were 62.9±9.2 in Δ*pefR versus* 5.1±1.7 in the WT strain).

The above *in vitro* and *in vivo* results indicate that PefR is a transcriptional repressor of both the *pefAB* and the *pefRCD* operons, by binding directly to the operator regions.

### PefR is an intracellular heme and PPIX sensor and regulator of *pef* expression

**Figure 4:**
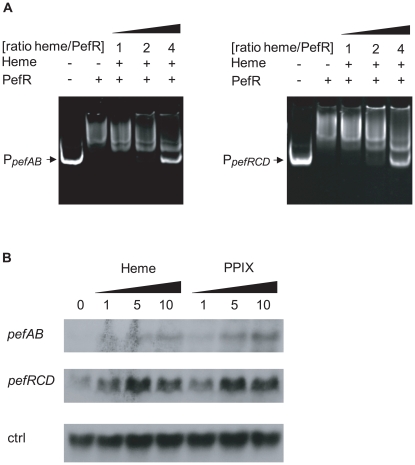
PefR is a heme- and PPIX-modulated repressor of *pefAB* and *pefRCD* loci. **A**. Gel mobility shift analysis of the effect of heme on PefR binding to *pefAB* (right) and *pefRCD* (left) promoter regions. 2 pmoles of P*_pefAB_* or P*_pefRCD_* fragments and 30 pmoles of PefR were mixed. Increased concentrations of heme were added in ratios indicated (keeping PefR constant). Assays were also performed with PPIX (Fig. S1A), giving similar results. On the two top panels, lanes 1–3 were juxtaposed to lanes 4–5, which were separated on the initial same gel. **B**. Northern blot analyses of *pefAB* and *pefRCD* expression in the presence of heme or PPIX in WT NEM316. Cultures were grown in the presence of 0, 1, 5 and 10 µM heme or PPIX, and harvested for total RNA extraction in early stationary phase. As probe efficiencies and times of exposure differ for each target RNA, differences between *pefAB* and *pefRCD* levels are not comparable. *ldhL* (*gbs0947*; ‘ctrl’) mRNA was used to control for RNA quantities.

A characteristic of MarR family regulators is their binding to effector molecules, which leads to induction or repression of their target genes [34]–[36]. Transcriptional fusions showed that the *pefAB* operon was induced in the presence of metalloporphyrins or PPIX (Fig. 2B). As PefR binds the two *pef* promoter regions, and appears to repress transcription, we hypothesized that PefR binding and repression are modulated by heme and PPIX. To test this *in vitro*, mobility shift assays between PefR and the *pefAB* or *pefRCD* promoter region DNA fragments were performed with and without heme or PPIX. For both DNA targets, PefR–DNA binding was alleviated in a heme or PPIX concentration-dependent manner (Fig. 4A, Fig. S1A). Iron had no effect on PefR-DNA binding (Fig. S1B).

Transcriptional fusion data showed that heme and PPIX up-regulate *pefAB* expression. To further confirm these results, we performed Northern blot experiments in the absence and presence of these molecules, using *pefAB* and *pefC* DNA as probes. Both heme and PPIX induced expression of the two *pef* operons (Fig. 4B). These *in vivo* results are in keeping with *in vitro* gel shift data showing that PefR repression is alleviated in the presence of these porphyrins. We conclude that heme and PPIX both modulate PefR repression of the *pefAB* and *pefRCD* operons.

### Expression of *hrtAB* as a function of heme and PPIX levels in *S. agalactiae*

**Figure 5:**
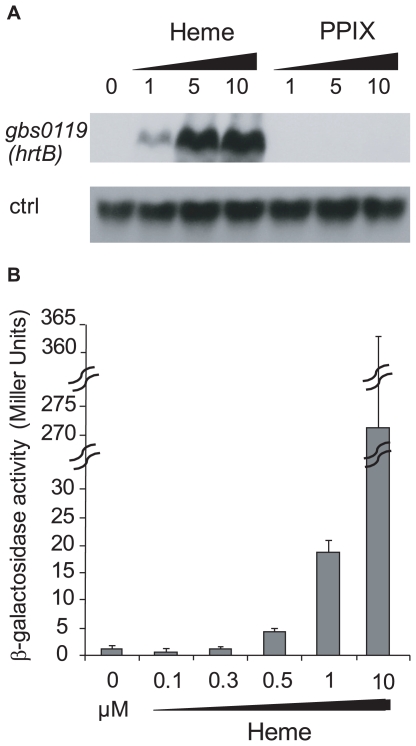
The *hrtAB* locus is induced by higher heme concentrations than *pefAB* or *pefRCD*, and is not induced by PPIX. **A**. Northern blot analyses of *gbs0119* expression in the presence of heme or PPIX in WT NEM316. Cultures were grown in the presence of 0, 1, 5 and 10 µM heme or PPIX, and harvested for total RNA extraction in early stationary phase. *ldhL* (*gbs0947*; ‘crtl’) mRNA was used as RNA quantity control. The hybridization was performed on the same membrane as that used in Fig. 4B. **B**. Expression analysis of P*_gbs0119_-lacZ* by determination of β-galactosidase activity in early stationary phase. *S. agalactiae* was grown in BHI liquid medium in the presence of different heme concentrations. Measurements were performed three or more times.

Genomic studies of *S. agalactiae* revealed the existence of an analog of HrtAB, a heme-regulated transport system initially characterized in *S. aureus* and shown to be involved in heme toxicity [37]. Gbs0119 and Gbs0120 showed 45% and 29% identity with HrtB and HrtA of *S. aureus* respectively. We used Northern blot experiments (Fig. 5A) and a *lacZ* promoter fusion, P*_gbs0119_-lacZ* (Fig. 5B), to assess the heme and PPIX concentrations needed to induce the *hrtAB* locus. Interestingly, *hrtAB* expression was low at heme concentrations below 1 µM, with strong induction at 10 µM; PPIX did not induce its expression, as was observed in *S. aureus*. Importantly, no *hrtAB* induction was observed at heme levels where *pefAB* was induced, i.e., between 0.1 and 0.5 µM heme (Fig. 2B). We conclude that the *pef* regulon is induced at heme concentrations below those needed for *hrtAB* induction, indicating that these functions are active under different conditions.

### Role of *pefAB* and *pefRCD* operons in porphyrin sensitivity

The above results led us to ask whether *pefAB* and *pefRCD* loci are involved in porphyrin efflux. Several *in vivo* approaches were developed to explore this question. We constructed in-frame *pefA* (encoding a putative drug:H+ antiporter) and *pefB* (encoding a membrane protein of unknown function) deletion mutants; as the two components of *pefCD* are predicted to encode a single ATP-dependent transporter, we generated a deletion removing both ORFs. Mutations were combined to inactivate both *pef* operons. Tests were also performed with the Δ*pefR* deletion mutant, in which expression of both *pef* loci were highly induced (Fig. 3C).

**Figure 6:**
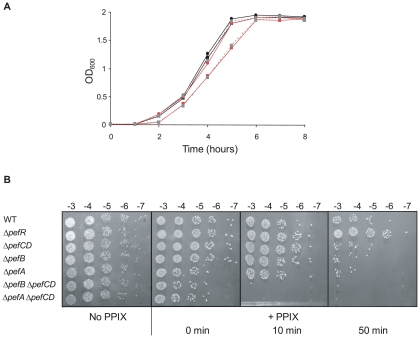
Δ*pefAB* and Δ*pefCD* mutants are affected in PPIX and heme sensitivity. **A**. Growth curves of WT and mutant strains in the presence of heme. Cells were grown aerobically in M17G medium supplemented with 0 or 1 µM heme. WT (black circle), WT +1 µM heme (black square), Δ*pefA* Δ*pefCD* (red circle), Δ*pefA* Δ*pefCD *+1 µM heme (red square), Δ*pefB* Δ*pefCD* (gray circle, dashed line) and Δ*pefB* Δ*pefCD *+1 µM heme (gray square, dashed line). Results shown are representative of 3 experiments. Growth retardation of Δ*pefA* Δ*pefCD* or Δ*pefB* Δ*pefCD* was more pronounced with 2 and 4 µM heme. **B**. Photosensitivity of WT and mutant *S. agalactiae* strains grown in the presence of PPIX. Cells were grown until early stationary phase in M17G medium supplemented or not with 10 µM PPIX. Serial 10-fold dilutions (exponent is indicated) were exposed to 0, 10, or 50 minutes visible light. Plates were photographed after 24 h incubation.

Sensitivity of Δ*pefR*, Δ*pefA* Δ*pefCD*, and Δ*pefB* Δ*pefCD* mutants to metalloporphyrins was evaluated by plate inhibition tests (Fig. S2). Both Δ*pefA* Δ*pefCD* and Δ*pefB* Δ*pefCD* mutants showed greater sensitivity than the WT strain to 1 nmole heme (Fig. S2A). Similar results were obtained using Gallium PPIX (GaPPIX; tested at 50 nmoles, Fig. S2B). We noted that Δ*pefA* Δ*pefCD* was more sensitive than Δ*pefB* Δ*pefCD*; this phenotypic difference might suggest an accessory role of PefB in PefA PefB function. The single Δ*pefA*, Δ*pefB*, Δ*pefC* or Δ*pefD* mutants gave little or no inhibition by these porphyrins (data not shown), suggesting a functional redundancy between the efflux systems encoded by these loci. Sensitivity of the Δ*pefR* mutant to heme and GaPPIX did not differ significantly from the WT. We further tested heme-mediated inhibition in liquid medium by growing WT and *pef* single and double mutants, and the Δ*pefR* mutant, in 0 or 1 µM heme (Fig. 6A and data not shown). The Δ*pefA* Δ*pefCD* and Δ*pefB* Δ*pefCD* mutants, but not the other tested strains, displayed a slight growth inhibition in the presence of heme. Addition of 2 µM or 4 µM heme exacerbates growth retardation of these mutants (data not shown). These results indicate the need for at least one of the Pef efflux systems to avoid heme toxicity.

To evaluate PPIX accumulation in *S. agalactiae* WT and mutant cells, we exploited its reactivity upon exposure to visible light; when excited by light, PPIX generates reactive oxygen species [38],[39]. The WT strain, and Δ*pefAB* Δ*pefCD* single and combined mutants, and the Δ*pefR* mutant, were grown with 10 µM PPIX and exposed to visible light for 0, 10 or 50 minutes. Viability of cells grown without PPIX and exposed to light was equivalent for all strains (Fig. 6B). Strikingly, inactivation of both putative pumps led to total mortality upon short light exposure (Fig. 6B). These results suggested that PPIX accumulates in *S. agalactiae* when *pefAB* and *pefCD* systems are inactivated. There were also marked differences between WT and Δ*pefR* sensitivity to PPIX after a longer (50 minute) light treatment. The Δ*pefR* mutant, which is up-regulated for *pefAB* and *pefCD* expression, showed essentially full viability, compared to a >10-fold drop for the WT strain. These data, showing heme and PPIX sensitivities of Δ*pefAB* Δ*pefCD* mutants, and lower PPIX sensitivity of the Δ*pefR* mutant give strong evidence for a role of the *pef* regulon in porphyrin efflux and intracellular homeostasis.

### Intracellular porphyrin availability in *S. agalactiae* WT and Δ*pefA* Δ*pefCD* strains

**Figure 7:**
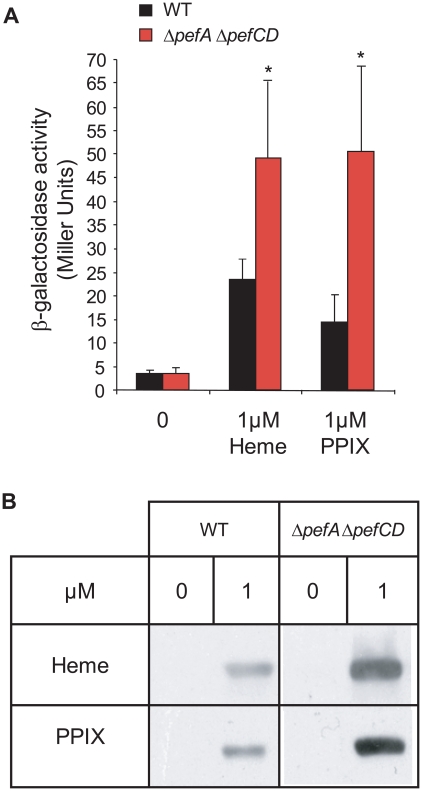
PPIX intracellular accumulation *in vivo*. **A**. Expression analysis of P*_pefA_-lacZ* by β-galactosidase activity determinations was performed in early stationary phase cells of WT and Δ*pefA* Δ*pefCD* strains. *S. agalactiae* and derivatives were grown in BHI liquid medium. Results represent the mean ± standard deviation from triplicate experiments. Asterisks denote statistically significant differences as determined by Student's t-test (p≤0.05). **B**. PPIX- or heme-dependent production of the *E. faecalis* catalase KatA in *S. agalactiae* WT, and Δ*pefA* Δ*pefCD* mutant strains. Cells were grown in M17G supplemented or not with 1 µM of PPIX or heme. The loading of equivalent amounts of protein was verified by Coomassie stained gels performed in parallel. KatA was detected in total *S. agalactiae* protein extracts by immunoblot assays. Results shown are representative of 3 experiments.

We used two approaches to evaluate differences in intracellular porphyrin in the WT *versus* Δ*pefA* Δ*pefCD* strain. First, we exploited the fact that heme- and PPIX- induce P*_pefA_-lacZ* (same as P*gbs_1753_-lacZ* above), to compare induction levels in the WT and Δ*pefA* Δ*pefCD* double mutant strains (Fig. 7A). In response to heme and PPIX addition, β-galactosidase activity was respectively about 2-fold and 3-fold increased in the Δ*pefA* Δ*pefCD* mutant compared to WT, suggesting that more heme and PPIX are available to activate the *pefA* (*gbs1753*) promoter in this mutant.

Second, we used an *in vivo* “heme and PPIX sensor”: the *E. faecalis* catalase KatA is degraded if not bound to a porphyrin molecule [19]. Intracellular availability of heme or PPIX was evaluated by comparing the relative stability of the *E. faecalis* KatA expressed in the WT strain and Δ*pefA* Δ*pefCD* and Δ*pefR* mutants (Fig. 7B and data not shown). Cells were grown without porphyrins or in 1 µM heme or PPIX, and KatA levels were followed in Western blots on cell lysates. Amounts of KatA in the WT and Δ*pefR* mutant strains were not significantly different (data not shown), which might reflect the limits of this reporter system. However, in the Δ*pefA* Δ*pefCD* mutant to which heme or PPIX was added, KatA showed pronounced stabilization, as expected for higher intracellular porphyrin levels in that strain. Results of both *in vivo* systems used above point to greater availability of porphyrins in the Δ*pefA* Δ*pefCD* mutant.

### Physiological impact of *pefAB* and *pefRCD* activities on *S. agalactiae* respiration and virulence

**Figure 8:**
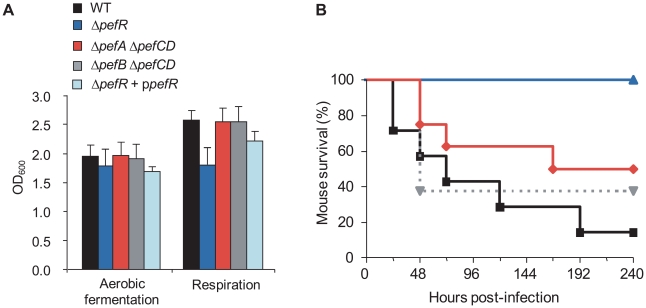
Physiological consequences of *pef* mutations. **A**. Impact on respiration metabolism of deregulating *pefAB pefCD* expression by *pefR* inactivation. *S. agalactiae* WT, Δ*pefA* Δ*pefCD*, Δ*pefB* Δ*pefCD*, Δ*pefR* and Δ*pefR* transformed with vector containing a wild-type copy of *pefR* (Δ*pefR* + p*pefR*) strains were grown in respiration-permissive conditions (i.e., 1 µM heme and 10 µM menaquinone). *S. agalactiae* respiration growth is characterized by a gain in cell density [17]. Absorbance (OD_600_) is shown on cultures after 22 h growth. Errors bars represent the standard deviation of three independent experiments. **B**. Impact on virulence of deregulating *pefAB pefCD* expression by *pefR* inactivation. Survival curves in adult mice infected with *S. agalactiae* WT (black square), Δ*pefA* Δ*pefCD* (red diamond), Δ*pefB* Δ*pefCD* (gray triangle, dashed line) or Δ*pefR* (blue triangle) strains. Differences in mortality between mice infected with the WT *versus* the Δ*pefR* mutant were statistically significant (p<0.001).

*S. agalactiae* takes up exogenous heme, which activates its membrane cytochrome *bd* quinol oxidase, and is needed for respiration metabolism; the shift to respiration increases cell density by at least 20% compared to aerobic fermentation growth [17]. We compared WT, Δ*pefR*, Δ*pefA* Δ*pefCD*, Δ*pefB* Δ*pefCD*, and the Δ*pefR* strain complemented by a plasmid-carried *pefR* gene (p*pefR*) for their capacity to grow in respiration conditions (Fig. 8A). The WT, Δ*pefA* Δ*pefCD*, Δ*pefB* Δ*pefCD*, and the complemented Δ*pefR* strain showed increased growth densities, indicative of respiration growth in these conditions. In contrast, the Δ*pefR* mutant was not augmented, suggesting a respiration defect of this strain. The *pefR::ISS1* insertional mutant gave the same results as the Δ*pefR* deletion strain (data not shown).

To confirm that the growth difference observed above was due to respiration metabolism, we performed the same growth experiments in the presence of 2-n-heptyl-4-hydroxyquinoline N-oxide (HQNO), which inhibits respiration but allows fermentation growth (data not shown; [17]). All tested strains except Δ*pefR* displayed lower growth densities in HQNO, which were comparable to those in aerobic fermentation; HQNO had no effect on cell density of the Δ*pefR* mutant. These results confirm that all strains tested above (Fig. 8A), except Δ*pefR*, activated respiration growth.

Respiration growth provokes an increase in oxygen consumption [17]. This property was examined to confirm the respiration-defect of Δ*pefR*. Oxygen consumption in aerobic fermentation growth by the WT, Δ*pefR*, and Δ*pefR* (p*pefR*) strains was measured as 20.3, 21.0, and 31.1 µM.min^−1^ per OD_600_ = 1 cells (Table 1), which in these conditions reflects cytoplasmic NADH oxidase activity [17]. In respiration-permissive conditions, oxygen consumption by the WT and Δ*pefR* (p*pefR*) strain was markedly higher (44.2 and 51.6 µM.min^−1^ respectively), but only moderately higher for the Δ*pefR* mutant (30.2 µM.min^−1^) (Table 1), further indicating the respiration-defect in the Δ*pefR* mutant. These results, together with the above studies, indicate that high PefAB and PefCD efflux activities impact on respiration by diminishing heme availability.

**Table 1:** Measurement of oxygen consumption according to metabolism in WT and Δ*pefR* strains.

Strains	Oxygen consumption (µM.min^−1^)	
	Aerobic fermentation	Respiration
WT	20.3±1.6	44.2±5.7
Δ*pefR*	21.0±6.6	30.2±4.8
Δ*pefR* + p*pefR*	31.1±3.2	51.6±0.3

*S. agalactiae* WT, Δ*pefR*, and Δ*pefR* complemented by a plasmid carrying a wild-type copy of *pefR* (Δ*pefR* + p*pefR*) were grown to late exponential phase. Oxygen consumption was measured on whole cells with a Clark-type oxygen electrode. Results represent the means ± SD of two (for Δ*pefR* + p*pefR*) or three experiments.

WT, Δ*pefAB* Δ*pefCD* and Δ*pefR* strains were tested in a mouse infection model (Fig. 8B). Virulence of the parental strain, and the Δ*pefA* Δ*pefCD* or Δ*pefB* Δ*pefCD* mutants was essentially the same. In contrast, the Δ*pefR* mutant (as well as the *pefR::ISS1* insertional mutant; data not shown) showed markedly attenuated virulence. Earlier findings showed that a *S. agalactiae* respiration-defective mutant is attenuated for virulence [17]. Reduced intracellular heme of *pefR* leads to a respiration deficiency, which can explain virulence attenuation of this mutant.

## Discussion

The present study uncovers a previously unknown system of porphyrin homeostasis in *S. agalactiae*, comprising two operons encoding distinct efflux pumps, controlled by a single intracellular regulator. Our results indicate that PefR, like numerous MarR-type proteins, acts as an intracellular sensor to regulate efflux of its ligands [40], [33], in this case, porphyrins. The range of PefR regulation is confined to the *pefAB* and *pefRCD* operons, as deduced from the presence of an IR within a 23 bp sequence uniquely upstream of these operons. This configuration is typical of MarR binding sites [33], [41], [42]. Expression of the *pefAB* and *pefCD* efflux loci limits *S. agalactiae* intracellular porphyrin availability; at high *pef* expression levels, *S. agalactiae* may be unable to activate respiration metabolism, and to cause septicemic infection in the animal host. The management of intracellular porphyrin levels by efflux pumps, and regulation by an intracellular sensor in *S. agalactiae* constitutes new information on heme and PPIX homeostasis strategies.

Homology searches revealed that 9 out of 10 species carrying *pefAB* genes also encode cytochrome *bd* quinol oxidases, and thus are genetically equipped (like *S. agalactiae*) to activate a respiration metabolism in the presence of heme, or heme and a menaquinone (Table S2). In contrast, the *pefRCD* operon seems to be specific to the *Streptococcus* genus, and is also present in species lacking the respiration genes. Among conditionally respiring bacteria, most characterized *S. agalactiae* isolates [43], and *Streptococcus uberis* encode the complete *pef* regulon. Since members of both these species share the same ecological niche (both are responsible for bovine intramammary infection [44], [45]), the presence of the complete *pef* regulon might conceivably arise from a genetic transfer event. However, this possibility is unlikely, as the identity between their *pef* ORFs (73% to 49%) is not higher than the 72% identity between these species at the genome level (Average Nucleotide Identity; calculated as in [46]). Conservation of the *pef* regulon may indicate a role in survival and/or infection in the particular biotopes of both these species.

Results of this study add heme and PPIX to the inventory of MarR interactants. Several MarR proteins were previously shown to bind to, and regulate efflux of lipophilic and planar molecules, such as antibiotics, aromatic aldehydes or fatty acids, usually as a means to limit cellular toxicity [47] [51]. PefR activity would expectedly be tuned to retain sufficient intracellular heme for functions such as respiration. Indeed, *pefAB* expression in cells grown with porphyrins is induced to only half the levels compared to a fully induced Δ*pefR* mutant (data not shown). This, and the fact that the Δ*pefR* mutant is respiration defective, supports the need for basal levels of intracellular heme. Moreover, the presence of two putative PefR binding sites upstream of *pefRCD* (*versus* one upstream of *pefAB*) might influence the differential regulation of the two loci; possibly, lower levels of *pefCD* expression could participate in limiting intracellular heme depletion.

PefAB and PefCD add to a handful of other systems described as being involved in porphyrin efflux. The PefAB and PefCD systems share common features with two efflux systems in eukaryotes that control porphyrin pools: Feline leukemia virus receptor C, FLVRC, is an MFS-family protein (like PefA) that exports cytoplasmic metalloporphyrins across an ion-proton gradient [52], [53]. The breast cancer resistance protein Bcrp/ABCG2 in humans is an ABC family transporter (like PefCD) that regulates eukaryotic intracellular heme levels under hypoxic conditions [54], [55]. Just two possible porphyrin efflux systems were described in bacteria, neither of which is an analog of PefAB or PefCD: In *E. coli*, TolC effluxes PPIX, possibly to facilitate its turnover after iron extraction from heme; neither its regulation, nor its capacity to efflux heme have been reported [5], [56]. In *S. aureus*, the *hrtAB* locus is regulated by heme, but not PPIX, and its inactivation provokes accrued heme sensitivity [28], [27]. The *S. agalactiae hrtAB* homologous system (*gbs0120 gbs0119*) is likely to have a similar role in limiting heme toxicity. In contrast, the *pef* regulon is activated by PPIX, and at heme concentrations that do not induce *hrtAB*.

As PefAB efflux relies on proton motive force (PMF) to export substrates, we speculate that activity would be stimulated by the greater PMF generated under respiration conditions, as shown in *L. lactis* [57], [17], [58]. Indeed, the PefAB system is conserved in streptococci and lactobacilli having the capacity to activate a respiration metabolism. As PefCD requires ATPase activity, its activity likely varies according to growth conditions [59], [60]. The need for both PefAB and PefCD activities in *S. agalactiae* may accommodate the different metabolic states encountered *in vivo*, e.g., anaerobic fermentation in abscess, aerobic fermentation in lungs, and respiration in sepsis.

**Figure 9:**
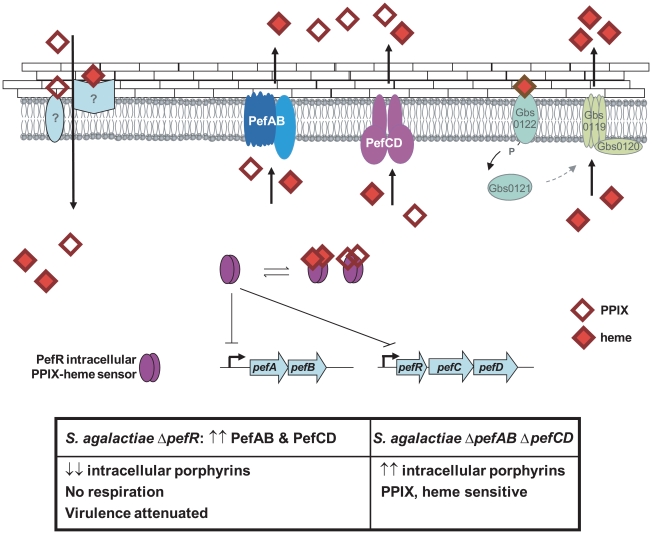
Model for *pefAB* and *pefCD* functions regulated by an intracellular sensor in *S. agalactiae*. Extracellular heme and PPIX may be internalized by as yet unknown transporters. By homology with a recently described system in *S. aureus* [27], heme, but not PPIX, may bind to a putative external two-component receptor protein Gbs0122, the homolog of *S. aureus* HssS, resulting in *gbs0119 gbs0120* (*hrtAB*) induction. Once inside the cell, free porphyrin molecules may encounter different binding proteins, including PefR. Apo-PefR binds to *pefAB* and *pefRCD* promoter regions to repress their expression. PefR-porphyrin binding releases PefR from DNA, to enable *pefAB* and *pefRCD* expression. The PefAB and PefCD loci mediate efflux of free heme and PPIX to avoid toxicity. Overexpression of PefAB and PefCD leads to heme depletion and respiration and virulence defects. Phenotypes of Δ*pefR versus* Δ*pefAB* Δ*pefCD* mutants are shown in the Table below.

A model for heme and PPIX homeostasis in *S. agalactiae*, based on the newly characterized Pef operons, is proposed (Fig. 9): PefR represses expression of *pefAB* and *pefRCD* operons. Heme and PPIX are assimilated by unknown mechanisms. Once intracellular, these molecules interact with PefR, which detaches from its binding sites to activate PefAB and PefCD efflux pumps. Activities of the two Pef systems may assure rapid adjustment of intracellular heme levels under different cell physiological conditions. Whether PPIX efflux by *pefAB pefCD* has a biological role in *S. agalactiae* as an enzymatic byproduct of iron capture as in *E. coli* [5] remains to be determined. At higher heme concentrations, HrtAB homologs Gbs0120 and Gbs0119 could be activated *via* the putative two-component system Gbs0122 (heme sensor) and Gbs0121 (regulator), as was demonstrated for HssRS in *S. aureus* [27]. This system would have a dominant role in protecting cells in a heme-rich environment, e.g., in conditions of massive degradation of host red blood cells; such a role is consistent with the strong induction of *hrtAB* (>200-fold) when heme levels are high (Fig. 5). Inactivation of *pefAB* and *pefCD* loci results in increased intracellular heme and PPIX pools, with consequences on growth and on heme-dependent enzyme activities; constitutive activity of these operons by *pefR* inactivation depletes these intracellular pools, resulting in respiration and virulence defects.

A role for respiration functions in virulence has been reported for diverse bacterial pathogens, including *Brucella abortus*, *Shigella flexnei*, *S. aureus* and *Mycobacterium tuberculosis*; unlike *S. agalactiae*, these bacteria biosynthesize heme and are autonomous for respiration [61] [64]. In *S. agalactiae*, respiration is part of the metabolic reprogramming that occurs during the requisite septicemic phase of infection, in heme-rich blood [17]. We suggest that a correlation between unavailability of heme due to high *pefAB* and *pefCD* activity, and the respiration defect explains virulence attenuation in the Δ*pefR* mutant. The use of efflux to regulate metabolic activity of a major bacterial pathogen has not been previously reported, and will lead to a better understanding of how bacteria deal with heme and other porphyrins in the variable conditions they encounter during infection.

## Materials and Methods

### Ethics statement

All animal experiments were performed in strict accordance with INRA institutional guidelines of good animal practice (Jouy-en-Josas, France), and approved by the Direction des Services Vétérinaires (accreditation number 78-63).

### Bacterial strains, growth conditions and plasmids

Strains and plasmids used in this study are described in Tables 2 and 3. NEM316, a *S. agalactiae* capsular serotype III strain was used as wild type (referred to as WT) and was isolated from a fatal case of septicemia [65]. *S. agalactiae* and its derivatives were cultivated at 37°C in M17 medium (Oxoid) supplemented with glucose (0.2% for pre-cultures and 1% for test cultures [referred to as M17G]). For β-galactosidase assays, *S. agalactiae* was grown in BHI (brain heart infusion, Difco Laboratories) supplemented with 0.8% glucose. Aerated cultures were grown under agitation at 200 rpm in a ratio of air space to liquid of approximately 5/1. Respiring cultures were grown under agitation in the presence of 1 µM hemin (from a stock solution of 10 mM hemin chloride, Fluka) and 10 µM vitamin K_2_ (a menaquinone; Sigma). *E. coli* was cultivated in LB medium (Luria-Bertani, Difco Laboratories) at 37°C with aeration by shaking at 180 rpm. Antibiotics were used as needed at the following concentrations: for *S. agalactiae*, 5 µg/ml erythromycin, 1000 µg/ml kanamycin, 4 µg/ml chloramphenicol, 5 µg/ml tetracycline; for *E. coli*, 100 µg/ml erythromycin, 20 µg/ml chloramphenicol.

**Table 2:** Strains used in this study.

Strain name	Main characteristics	Reference
*E. coli*		
TOP10	F- *mcr*A Δ(*mrr-hsd*RMS-*mcr*BC) Φ80*lac*ZΔM15 D*lac*X74 *rec*A1 *ara*D139 Δ(*ara-leu*)7697 *gal*U *gal*K *rps*L (Str^R^) *end*A1 *nup*G	Invitrogen
TG1repA^+^	*supE* hsd-5 *thi* Δ(*lac-proAB*)F′(*traD36 proAB^+^ lacI^q^ lacZΔM15*) *repA* ^+^	[71]
BL21 Star (DE3)	F^−^ *omp*T *hsd*S_B_(r_B_^−^m_B_^−^) *gal dcm* (DE3)	Invitrogen
*S. agalactiae* NEM316		
NEM316	Serotype III isolated from neonatal blood culture	[65]
NEMJ11	NEM316 Δ*pefA*. In-frame deletion of amino acid (aa) positions 36–433 of PefA.	This study
NEMJ12	NEM316 Δ*pefB*. In-frame deletion of aa positions 26–211 of PefB.	This study
NEMJ13	NEM316 Δ*pefC* Δ*pefD*. Double deletion of aa positions 133–605 of PefC and positions 1–419 of PefD.	This study
NEMJ14	NEM316 Δ*pefR*. In-frame deletion of aa positions 24–145 of PefR.	This study
NEMJ15	NEM316 Δ*pefA* Δ*pefCD*	This study
NEMJ16	NEM316 Δ*pefB* Δ*pefCD*	This study
NEMB08	NEM316 *gbs1402*::IS*S1*. IS*S1* insertion between codon positions 81 and 82 aa of the *gbs1402* ORF.	This study

**Table 3:** Plasmids used in this study.

Plasmid name	Main characteristics	Reference
pTCV-*lac*	Conjugative *E. coli*-Gram-positive bacteria shuttle plasmid with β-galactosidase reporter construct. Em^R^ Km^R^.	[32]
pTCV-J11	P_gbs1753_-*lacZ* or P_pefA_-*lacZ. gbs1753* (*pefA*) promoter region cloned into pTCV-*lac*. Em^R^ Km^R^.	This study
pTCV-J21	P_gbs0119_-*lacZ*. *gbs0119* promoter region cloned into pTCV-*lac*. Em^R^ Km^R^.	This study
pG+host8::IS*S1*	Vector for insertional mutagenesis. Tet^R^.	[69]
pG+host5	Temperature sensitive vector used in Gram-positive bacteria. Ery^R^.	[70]
pRC2	Cloning vector for complementation. Cm^R^	[72]
pRC2-J14	pRC2 plasmid containing *pefR* gene, referred as p*pefR*. Cm^R^.	This study
pET100/D-TOPO	Cloning vector. Amp^R^	Invitrogen
pET100-J14	Expression N-terminal His tagged PefR. Amp^R^	This study
pLUMB5	P_aphA-3_-*katA*-His_6_. Expression *E. faecalis* KatA. Kanamycin resistance promoter. Cm^R^	[19]

### RNA extraction, Northern blot and RACE PCR

RNA was extracted from *S. agalactiae* cultures as described [66]. Cells from 20 ml culture with an OD_600_ of 0.5∼0.8 (exponential growth phase) or OD_600_∼1.5 were harvested by centrifugation at 6000×g during 10 min. Total RNA was extracted with a guanidine isothycionate and phenol-chloroform step [67], using the TRIzol Reagent (Invitrogen).

For Northern blot analysis, RNA samples (30 µg) were mixed with an equal volume of glyoxal load dye (Ambion) and were electrophoretically separated on a 0.9% agarose glyoxal gel [68]. RNA samples were transferred to a Biodyne B membrane (Pall) according to manufacturer's instructions. Hybridization and detection were performed using ECL direct nucleic acid labeling and detection systems (Amersham). A ∼500 base pair (bp) DNA labeled fragment of each ORF tested was used as probe. Primers used for probe generation are shown in Table S3.

The *pefA* transcriptional start was mapped using a 5′/3′ rapid amplification cDNA ends (RACE) kit, second generation (Roche Applied Science) according to supplier's instructions. Mapping was realized with RNA extracted from respiring cells, using primers: 5′TAGATGTAGGTGCTAACGTCG3′ and 5′CTTGTTGAGCCGTTGACAACG3′.

### β-galactosidase assays

Plasmid pTCV-*lac* is a low copy number plasmid used to evaluate promoter activities in *S. agalactiae* [32]. A DNA fragment containing *gbs1753* promoter was PCR-amplified with primers 5′GCGTAGAATTCATTAAATGGAG3′, containing an *Eco*RI site (underlined) and 5′TATCTCGGATCCCTATTTCTGAT3′, containing a *Bam*HI site (underlined). After digestion of the amplified fragment by *Eco*RI and *Bam*HI, the *gbs1753* promoter was cloned into plasmid pTCV-*lac*, resulting in plasmid pTCV-J11 carrying the P*_gbs1753_-lacZ* (or P*_pefA_-lacZ*) fusion. The plasmids pTCV-*lac* and pTCV-J11 were subsequently transformed into *S. agalactiae* by electroporation and recombinant clones were obtained by erythromycin selection. The same strategy was performed to generate a P*_gbs0119_*-*lacZ* fusion using the primers 5′ATACGCCAGAATTCTCGGCGAC3′ and 5′CCTTGGATCCTTTGATGTGAAC3′, giving rise to plasmid pTCV-J21.

### Mutagenesis and screening conditions

Random insertional mutagenesis was performed on *S. agalactiae* with pG+host8::IS*S1* basically as described [69]. Both P*_gbs1753_*-*lacZ* (reporter for *gbs1753* transcription) and pG+host8::IS*S1* plasmids were established in *S. agalactiae*. The strain was grown at 30°C with kanamycin for 2.5 h. The temperature was then shifted to 37°C for 2.5 h and plated on M17G with erythromycin, kanamycin and 80 µg/ml X-gal (5-bromo-4-chloro-3-indolyl-beta-D-galactopyranoside, BioMerieux), to obtain ∼100 CFU per plate. After 24 h incubation at 37°C, deep blue colonies were selected. Stable IS*S1* mutants were isolated by growth at 30°C without erythromycin as described [70]. The flanking chromosomal DNA was sequenced using primer 5′AGGGCATGGAAACAATTCGAGG3′. From the 5000 mutants we screened, 7 were in the *pefR* locus. Stable IS*S1* mutants were then retested for *gbs1753* up-regulation.

### Gbs1402 (PefR) overexpression and purification

The *gbs1402* ORF was amplified by PCR from *S. agalactiae* NEM316 using primers 5′CACCATGGAGAATCCTCTTCAAAA3′ and 5′TAATCAAAATCTTCCATCGCC3′. This amplified fragment was cloned into the *E. coli* expression plasmid Champion pET100 directional TOPO vector (Invitrogen) and transformed into *E. coli* TOP10. After plasmid verification by sequencing, the plasmid pET100-J14 was electroporated in *E. coli* BL21 (DE3). Protein expression was induced at an OD_600_ = 0.6 by addition of 1 mM IPTG for 2 h. Cells were harvested by centrifugation at 6000 rpm for 10 min and resuspended in 10 ml binding buffer (Tris HCl 50 mM pH 8, NaCl 300 mM, Imidazole 50 mM). Cells were broken by shaking with glass beads in a Fast Prep apparatus (MP Biomedicals). The lysate was centrifuged at 14000 rpm for 15 min at 4°C. The recombinant His-tagged fusion protein was purified by nickel affinity chromatography using His-Select affinity (Sigma) equilibrated with binding buffer according to supplier's instructions. The elution was carried out with binding buffer containing 300 mM imidazole. All fractions were collected and analyzed by SDS-PAGE. Purified proteins were dialyzed against 50 mM Tris HCl pH 8, 80 mM NaCl and 6.25% glycerol. Protein concentrations were determined by the Lowry assay (Bio-Rad).

### Immunoblotting analysis

Strains harboring P_aphA-3_-*katA*-His_6_ plasmid were incubated overnight under static conditions with or without 1 µM heme or PPIX. Cell growth was harvested by centrifugation at 6000×g during 10 min. Cells were broken by shaking with glass beads in a Fast Prep apparatus (MP Biomedicals). Immunoblotting analysis was done with rabbit anti-KatA antiserum as described, and kindly provided by Dr. L. Hederstedt [19]. Detection was performed using ECL Plus Western blotting detection reagents (GE Healthcare).

### Electrophoretic mobility shift assay (EMSA)

A 281 bp DNA fragment containing the *gbs1753* promoter region and a 248 bp DNA fragment containing the *gbs1402* promoter region were generated by PCR from genomic DNA of *S. agalactiae* and purified using the PureLink PCR Purification Kit (Invitrogen). Binding assays with PefR were carried out using a binding buffer (5% glycerol, 20 mM Tris HCl pH 8, 50 mM KCl, 0.2 mM MgCl_2_, 1 mM EDTA, 0.2 mM DTT, 0.3 µM BSA) in a final volume of 15 µl at 37°C. Reaction mixtures were incubated at 37°C for 20 min, after which they were analyzed by gel electrophoresis on an 8% polyacrylamide gel in TBE 1× buffer, stained with ethidium bromide. Reactions were carried out in the presence of a 116 bp non-specific competitor DNA (internal coding *gbs1753* sequence) where specified. Where tested, heme or PPIX were added 20 min after protein and DNA components. Gel shift experiments were performed with two independent batches of purified PefR.

### Construction of Δ*gbs1753* (Δ*pefA*), Δ*gbs1752* (Δ*pefB*), Δ*gbs1401* Δ*gbs1400* (Δ*pefCD*) *and* Δ*gbs1402* (Δ*pefR*) mutants and p*pefR* plasmid construction

In-frame *pefA*, *pefB*, *pefCD*, and *pefR* deletion mutants were constructed using the strategy described below. Briefly, the two regions flanking the locus to be deleted were independently amplified by PCR (see Table S3 for primers). Amplified fragments were digested by *Hin*dIII or *Eco*RV enzyme and ligated to each other. The resulting fragments were amplified by PCR, digested by *Eco*RI enzyme and cloned into the thermosensitive shuttle plasmid pG+host5. Electroporation of *S. agalactiae* strains and allelic exchange were done as described [70]. In-frame deletions were confirmed by PCR and sequence analysis. The *pefR* gene was amplified (Table S3 for primers), and cloned using *Eco*RI and *Bam*HI on fragment ends, onto plasmid pRC2 cut with the same enzymes; the resulting plasmid, pRC2-J14, is referred to as p*pefR* in Results.

### PPIX and metalloporphyrin sensitivity tests

Stock solutions (10 mM) of PPIX, GaPPIX, ZnPPIX, and ZnMPIX (Frontier Scientific) were prepared in DMSO. PPIX sensitivity of *S. agalactiae* NEM316 and its derivatives was examined on early stationary growing cells (OD_600_ = 1.6). For PPIX light sensitivity tests, serial dilutions were spotted on plates and exposed to visible light for 0, 10 or 50 minutes. A 500 W halogen lamp with a glass lid placed at a distance of 55 cm was used as light source. Metalloporphyrin sensitivity tests on plates were performed as described [12]: Briefly, 1∶100 dilutions of stationary-phase cultures were prepared in M17G soft (0.7%) agar, and poured over M17G plates. Metalloporphyrin (heme, 5 µl of a 0.2 mM solution; GaPPIX, 5 µl of a 10 mM solution) was spotted onto the plates. All plates were incubated for 20 h at 37°C and then photographed.

### Oxygen consumption.measurements

Oxygen consumption was determined as described [17]. Briefly, *S. agalactiae* WT and Δ*pefR* late exponential phase cultures, prepared in aerobic fermentation or respiration conditions, were washed twice with PBS at 4°C and resuspended in the same buffer to obtain a 1 ml bacterial suspension at OD_600_ = 1.0. Oxygen consumption was followed with a Clark-type oxygen electrode (Liquid-Phase Oxygen electrode unit DW1, Hansatech instruments). The maximum oxygen consumption rate was measured following the addition of 10 mM glucose.

### Mouse virulence assay

Pathogen-free 6 week-old Swiss CD1 mice (Charles River laboratories) were infected intravenously with *S. agalactiae* NEM316 or derivatives. Groups of 7–8 mice were anesthetized with ketamine (100 µg/g, Merial) and xylazine (12 µg/g, Bayer) and were inoculated in the eye vein with 10^8^ CFU. Bacterial counts were determined in parallel by plating serial dilutions in saline buffer on M17G. Cells were harvested from mid-log phase and washed in a solution of NaCl 0.9%. Mortality was observed over a 10-day period. Animal experiments were performed in triplicate.

### Bioinformatics

To find the conserved sequence between *pef* promoter regions, sequence were aligned with ClustalW and manually refined. A search for the 23 bp consensus in *S. agalactiae* NEM316 genome was done by visual inspection, and using BlastN program with EXPECT threshold set at 100. Pef protein homologs were identified using Genome Region Comparison, http://cmr.jcvi.org/cgi-bin/CMR/CmrHomePage.cgi, and/or were retrieved from genomic Blast databases, http://blast.ncbi.nlm.nih.gov/Blast.cgi/, using BlastP program with an E-value threshold set at 0.01.

## Supporting Information

Figure S1 A. PPIX displaces PefR from its DNA target. Gel mobility shift analysis of the effect of PPIX on PefR binding to the *pefAB* (right) and *pefRCD* (left) promoter regions. Samples contained 2 pmoles of *P_pefAB_* or *P_pefRCD_* fragment and 30 pmoles of PefR, to which were added either sample buffer, 120 pmoles (left) or 240 pmoles (right) of PPIX. On the two top panels, lanes 1–2 were juxtaposed to lane 3, which were separated on the initial same gel. B. Heme, but not iron, displaces PefR from its DNA target. Samples contained 2 pmoles of *P_pefAB_* fragment and 32 pmoles of PefR, to which were added either sample buffer, 160 pmoles heme, or 160 pmoles iron. Lanes 1–2 were juxtaposed to lane 3–4, which were separated on the initial same gel.
(1.51 MB EPS)

Figure S2 Differential heme (FePPIX) and GaPPIX sensitivity of the *pef* mutants. Stationary phase cultures of WT, Δ*pefR*, Δ*pefA* Δ*pefCD* or Δ*pefB* Δ*pefCD* strains were spread on M17G plates and 1 nmole of heme (A) or 50 nmoles of GaPPIX (B) were pipetted directed onto plates. Plates were incubated 24 hours, and then photographed. Representative results of at least 3 experiments are shown.
(1.40 MB EPS)

Table S1 Transcriptome analysis in respiration *versus* aerobic fermentation conditions. Cells were grown in M17 medium with 1% glucose, supplemented or not with a mixture of 10 µM vitamin K_2_ (a menaquinone) and 48 µg/ml hemoglobin as heme source. Cells were harvested at OD_600_ = 0.3 for RNA extraction. Total RNA was extracted and analyzed by hybridization on NEM316 derived whole genome DNA macroarray as described [66]. Fold change in expression is the mean of two independent macroarray experiments. Shown are results of *S. agalactiae* genes whose expression was induced or repressed at least 2-fold in respiration compared to aeration conditions. Genes further studied in this work are in bold. Gene assignments are according to the Sagalist web site (http://genolist.pasteur.fr/SagaList/) and BLAST searches (http://blast.ncbi.nlm.nih.gov/Blast.cgi/).
(0.09 MB DOC)

Table S2 Distribution of *pef* regulon and cytochrome genes among Lactobacillales. * Reorganization of gene or domain order; # frameshift mutation in *pefA* homologous gene. In bold, species containing the complete *pef* regulon; ** Imperfect repeats, characteristic of MarR DNA binding sites, are present in respective *pefAB* and *pefRCD* promoter regions of this strain; *** *gbs1401* and *gbs1400* ORFs are missing in *S. agalactiae* 2603V/R [43].
(0.06 MB DOC)

Table S3 Primers used in this study.
(0.05 MB DOC)
