# Supplementary material for: Two Coregulated Efflux Transporters Modulate Intracellular Heme and Protoporphyrin IX Availability in Streptococcus agalactiae
Source: PLoS Pathog. 2010 Apr 22;6(4):e1000860. doi: 10.1371/journal.ppat.1000860 (PMC2858704; doi:10.1371/journal.ppat.1000860)
Supplement: Table S1 — Transcriptome analysis in respiration versus aerobic fermentation conditions. Cells were grown in M17 medium with 1% glucose, supplemented or not with a mixture of 10 µM vitamin K2 (a menaquinone) and 48 µg/ml hemoglobin as heme source. Cells were harvested at OD600 = 0.3 for RNA extraction. Total RNA was extracted and analyzed by hybridization on NEM316 derived whole genome DNA macroarray as described [66]. Fold change in expression is the mean of two independent macroarray experiments. Shown are results of S. agalactiae genes whose expression was induced or repressed at least 2-fold in respiration compared to aeration conditions. Genes further studied in this work are in bold. Gene assignments are according to the Sagalist web site (http://genolist.pasteur.fr/SagaList/) and BLAST searches (http://blast.ncbi.nlm.nih.gov/Blast.cgi/). (0.09 MB DOC) [file ppat.1000860.s003.doc]

**Table S1**. Transcriptome analysis in respiration *versus* aerobic fermentation conditions.

| ***S. agalactiae* ORF** | **Gene name** | **Putative function** | **Fold change** |
| --- | --- | --- | --- |
| *gbs0018* |  | Aromatic amino acid aminotransferase | 2.1 |
| *gbs0019* | *recO* | DNA repair protein | 2.5 |
| *gbs0023* | *purC* | Phosphoribosylaminoimidazole-succinocarboxamide synthase | 4.0 |
| *gbs0025* | *purF* | Amidophosphoribosyltransferase | 3.3 |
| *gbs0026* | *purM* | Phosphoribosylformylglycinamide cyclo-ligase | 2.8 |
| *gbs0027* | *purN* | Phosphoribosylglycinamide formyltransferase | 2.7 |
| *gbs0028* |  | GNAT family acetyltransferase | 6.0 |
| *gbs0029* | *purH* | Bifunctional phosphoribosylaminoimidazolecarboxamide formyltransferase/IMP cyclohydrolase | 3.6 |
| *gbs0044* |  | Phosphoribosylaminoimidazole carboxylase ATPase subunit | 3.0 |
| *gbs0045* |  | Hypothetical protein | 4.5 |
| *gbs0046* |  | Hypothetical protein | 2.4 |
| *gbs0047* |  | Adenylosuccinate lyase | 2.5 |
| *gbs0057* | *rbsJ* | Ribosomal protein S10 | -2.1 |
| *gbs0076* | *rpmD* | Ribosomal protein L30 | -2.7 |
| *gbs0331* | *fabH* | Beta-ketoacyl-ACP synthase III | -2.3 |
| *gbs0352* |  | Xanthine/uracil permease family protein | 2.6 |
| *gbs0440* |  | Lipoprotein of unknown function | 2.8 |
| *gbs0539* |  | Phosphoglucomutase/phosphomannomutase family protein | 2.1 |
| *gbs0553* | *pyrDA* | Dihydroorotate dehydrogenase 1A | 2.0 |
| *gbs0789* |  | Oxalate:formate antiporter | 2.4 |
| *gbs0806* |  | Conserved hypothetical protein | 3.9 |
| *gbs0809* | *licT* | Transcriptional antiterminator | 3.4 |
| *gbs0921* |  | Methionine sulfoxide reductase B | 2.0 |
| *gbs0939* | *coaA* | Pantothenate kinase | -2.1 |
| *gbs1062* |  | Conserved hypothetical protein | -2.3 |
| *gbs1065* |  | Hypothetical protein | -2.0 |
| *gbs1068* |  | FtsK/SpoIIIE family protein | -3.1 |
| *gbs1070* |  | Conserved hypothetical protein | -3.4 |
| *gbs1071* |  | Hypothetical protein | -2.2 |
| *gbs1072* |  | Hypothetical protein | -2.1 |
| *gbs1074* |  | Conserved hypothetical protein | -2.7 |
| *gbs1075* |  | Conserved hypothetical protein | -3.9 |
| *gbs1077* | *carB* | Carbamoyl-phosphate synthase | -2.2 |
| *gbs1078* | *carA* | Glutaminase of carbamoyl-phosphate synthase | -4.6 |
| *gbs1079* | *pyrB* | Aspartate carbamoyltransferase catalytic subunit | -6.4 |
| *gbs1080* | *pyrC* | Dihydroorotase | -3.0 |
| *gbs1081* | *pyrE* | Orotate phosphoribosyltransferase | -3.1 |
| *gbs1082* | *pyrF* | Orotidine 5'-phosphate decarboxylase | -4.4 |
| *gbs1116* |  | Xanthine permease | 2.7 |
| *gbs1154* |  | Guanosine 5'-monophosphate oxidoreductase | 3.0 |
| *gbs1210* |  | Thioesterase family protein | -2.0 |
| *gbs1211* |  | Uracil permease | -5.9 |
| *gbs1229* |  | DNA topoisomerase IV, subunit B | -2.0 |
| *gbs1319* |  | Hypothetical protein | 2.5 |
| *gbs1373* |  | Hypothetical protein | 2.5 |
| *gbs1478* |  | PI-2a ancillary protein 1 | -2.0 |
| *gbs1587* |  | Manganese ABC transporter, permease protein | 2.0 |
| *gbs1588* |  | Manganese ABC transporter, ATP-binding protein | 2.2 |
| *gbs1612* | *tehB* | Tellurite resistance protein | 2.7 |
| *gbs1628* |  | Branched-chain amino acid ABC transporter, ATP-binding protein | 2.4 |
| *gbs1629* |  | Branched-chain amino acid ABC transporter, ATP-binding protein | 2.1 |
| ***gbs1752*** | ***pefB*** | **Conserved hypothetical protein** | **5.1** |
| ***gbs1753*** | ***pefA*** | **Drug:H+ antiporter** | **2.4** |
| *gbs1763* |  | PAP2 family protein | 2.7 |
| *gbs1799* |  | Similar to low specificit L-threonine aldolase | 2.2 |
| *gbs1859* | *purA* | Adenylosuccinate synthase | 2.2 |
| *gbs2013* |  | Major facilitator superfamily transporter | -5.4 |
| *gbs2053* |  | Cold shock protein | 2.0 |

Cells were grown in M17 medium with 1% glucose, supplemented or not with a mixture of 10 µM vitamin K_2_ (a menaquinone) and 48 µg/ml hemoglobin as heme source. Cells were harvested at OD_600_ = 0.3 for RNA extraction. Total RNA was extracted and analyzed by hybridization on NEM316 derived whole genome DNA macroarray as described [66]. Fold change in expression is the mean of two independent macroarray experiments. Shown are results of *S. agalactiae* genes whose expression was induced or repressed at least 2-fold in respiration compared to aeration conditions. Genes further studied in this work are in bold. Gene assignments are according to the Sagalist web site (<http://genolist.pasteur.fr/SagaList/>) and BLAST searches (<http://blast.ncbi.nlm.nih.gov/Blast.cgi/>).
