# Supplementary material for: Two Coregulated Efflux Transporters Modulate Intracellular Heme and Protoporphyrin IX Availability in Streptococcus agalactiae
Source: PLoS Pathog. 2010 Apr 22;6(4):e1000860. doi: 10.1371/journal.ppat.1000860 (PMC2858704; doi:10.1371/journal.ppat.1000860)
Supplement: Table S2 — Distribution of pef regulon and cytochrome genes among Lactobacillales. * Reorganization of gene or domain order; # frameshift mutation in pefA homologous gene. In bold, species containing the complete pef regulon; ** Imperfect repeats, characteristic of MarR DNA binding sites, are present in respective pefAB and pefRCD promoter regions of this strain; *** gbs1401 and gbs1400 ORFs are missing in S. agalactiae 2603V/R [43]. (0.06 MB DOC) [file ppat.1000860.s004.doc]

**Table S2**. Distribution of *pef* regulon and cytochrome genes among Lactobacillales.

| **Species** | ***cydAB*** | ***pefAB*** | ***pefRCD*** |
| --- | --- | --- | --- |
| *Enterococcus faecalis* V583 | *EF_2061-2060* | *EF_0420-0419* |  |
| *Enterococcus faecium* DO |  |  |  |
| *Lactobacillus acidophilus* NCFM |  | *LBA1446-1447* |  |
| *Lactobacillus brevis* ATCC 367 | *LVIS_1642-1641* | *LVIS_1724-1723* |  |
| *Lactobacillus casei* ATCC 334 | *LSEI_2205-2204* | *LSEI_0880-0881* |  |
| *Lactobacillus delbrueckii* subsp*. bulgaricus* ATCC 1842 |  |  |  |
| *Lactobacillus gasseri* ATCC 33323 | *LGAS_1841-1842* | *LGAS_0725-0724* |  |
| *Lactobacillus johnsonii* NCC 533 | *LJ_1810-1811* | *LJ_1576-1577* |  |
| *Lactobacillus plantarum* WCFS1 | *lp_1125-1126* | *lp_0991-0990* |  |
| *Lactobacillus reuteri* DSM 20016 | *Lreu_0505-0506* | *Lreu_1519-1520* |  |
| *Lactobacillus sakeii 23K* |  | *LSA0420****^#^****-0418* |  |
| *Lactobacillus salivarius* UCC118 | *LSL_1032-1031* | *LSL_0076-0077* |  |
| *Lactococcus lactis* subsp*. cremoris* SK11 | *LACR_0737-0738* | *LACR_1383-1382* |  |
| *Lactococcus lactis* subsp*. lactis* Il1403 | *L107762-109201* | *L132251-133825* |  |
| *Leuconostoc mesenteroides* ATCC 8293 | *LEUM_0560-0561* | *LEUM_1880-1879* |  |
| *Oenococcus oeni* PSU | *OEOE_1837-1836* | *OEOE_1378-1377** |  |
| ***Streptococcus agalactiae* NEM316****, ******* | ***gbs1787-1786*** | ***gbs1753-1752*** | ***gbs1402-1400*** |
| *Streptococcus dysgalactiae* subsp*. equisimilis* GGS_124 | *SDEG_0120-0121* |  | *SDEG_1978-1976* |
| *Streptococcus equi* subsp*. zooepidemicus* MGCS10565 |  |  | *Sez_0219-0221* |
| *Streptococcus gordonii* Challis NCTC7868 |  |  | *SGO_1752-1750* |
| *Streptococcus mitis* NCTC12261 |  |  | *SMT1859-1861** |
| *Streptococcus mutans* UA159 |  |  | *SMU_921-923* |
| *Streptococcus pneumoniae* TIGR4 |  |  | *SP_1920-1918** |
| *Streptococcus pyogenes* MGAS315 |  |  | *spyM3_0163-0165* |
| *Streptococcus sanguinis* SK36 |  |  | *SSA_0460-0462* |
| *Streptococcus suis* 05ZYH33 |  |  | *SSU05_2039-2037* |
| *Streptococcus thermophilus* LMG18311 |  |  | *stu0432-0434* |
| ***Streptococcus uberis* 0140J**** | ***SUB0104-0105*** | ***SUB1642-1643*** | ***SUB1690-1688*** |

* Reorganization of gene or domain order; # frameshift mutation in *pefA* homologous gene. In bold, species containing the complete *pef* regulon; ** Imperfect repeats, characteristic of MarR DNA binding sites, are present in respective *pefAB* and *pefRCD* promoter regions of this strain; *** *gbs1401* and *gbs1400* ORFs are missing in *S. agalactiae* 2603V/R [43].
