# Supplementary material for: Two Coregulated Efflux Transporters Modulate Intracellular Heme and Protoporphyrin IX Availability in Streptococcus agalactiae
Source: PLoS Pathog. 2010 Apr 22;6(4):e1000860. doi: 10.1371/journal.ppat.1000860 (PMC2858704; doi:10.1371/journal.ppat.1000860)
Supplement: Table S3 — Primers used in this study. (0.05 MB DOC) [file ppat.1000860.s005.doc]

**Table S3.** Primers used in this study.

| **Primer name** | **Comments** | **Sequence 5’->3’** |
| --- | --- | --- |
| pefR.I1 | Δ*pefR* mutant | CATAATA**GAATTC**CCAGCGAC |
| pefR.I2 | Δ*pefR* mutant | CATCAT**AAGCTT**TTGCATAACGA |
| pefR.I3 | Δ*pefR* mutant | GCGATGG**AAGCTT**TTGATTAAC |
| pefR.I4 | Δ*pefR* mutant | AGCTGAGT**GAATTC**GTTAGTT |
| pef1A.I1 | Δ*pefA* mutant | GCTGACC**GAATTC**TTGATCTCG |
| pef1A.I2 | Δ*pefA* mutant | GCCGTTCCC**AAGCTT**GTCTGA |
| pef1A.I3 | Δ*pefA* mutant | GCATTGCTTTC**AAGCTT**TGCTCAA |
| pef1A.I4 | Δ*pefA* mutant | CATCAGC**GAATTC**GTAGCTAGC |
| pef1B.I1 | Δ*pefB* mutant | GTTAGTC**GAATTC**CTGGAGCTGT |
| pef1B.I2 | Δ*pefB* mutant | ACCTAG**GATATC**CCGTAGAGC |
| pef1B.I3 | Δ*pefB* mutant | GCTATG**GATATC**AAGACATTAGC |
| pef1B.I4 | Δ*pefB* mutant | TATGATAAGA**GAATTC**AGTTTCAA |
| pef2A.I1 | Δ*pefCD* mutant | CATCTC**GAATTC**TGTAGCTTC |
| pef2A.I2 | Δ*pefCD* mutant | GTAAC**AAGCTT**CATTCCCAT |
| pef2B.I3 | Δ*pefCD* mutant | TCAG**AAGCTT**GCTATTGTAGG |
| pef2B.I4 | Δ*pefCD* mutant | CCTTC**GAATTC**TCTCTTAAGCT |
| pVS.pefR.1 | Δ*pefR* complementation | CATTT**GGATCC**TACTATCATTTACA |
| pVS.pefR.2 | Δ*pefR* complementatio*n* | TTTC**GAATTC**ATCCTATTATCC |
| pef1.N1 | *pefAB* northern blot | ATACTGCTCGTCAAATTGCT |
| pef1.N2 | *pefAB* northern blot | AATCATGAGACCAAATGGA |
| pef2.N1 | *pefRCD* northern blot | CATGTGATGGATTATTCTGA |
| pef2.N2 | *pefRCD* northern blot | TCATAAGCGACATCAATCTA |
| ldh.N1 | *ldh* northern blot | aagttatcctcgttggtgat |
| ldh.N2 | *ldh* northern blot | gatgtatgcgtgaactgaac |
| gbs0119.N1 | *gbs0119* northern blot | AATAACCCAGCTCAAACCTA |
| gbs0119.N2 | *gbs0119* northern blot | AAACAGTGCCAATATGATGA |
| pef1.GS1 | *pefAB* promoter gel shift | GCGTAGAAGTCATTAAATGGAG |
| pef1.GS2 | *pefAB* promoter gel shift | TATCTCCAATCTCTATTTCTGAT |
| pef2.GS1 | *pefRCD* promoter gel shift | atttgtattttactatcatttac |
| pef2.GS2 | *pefRCD* promoter gel shift | gattctccataataatactcc |
| ctrl.GS1 | Control gel shift | GATCACCTTATTGTTGATTGC |
| ctrl.GS2 | Control gel shift | CTTGTTGAGCCGTTGACAACG |
